# On InChI and evaluating the quality of cross-reference links

**DOI:** 10.1186/1758-2946-6-15

**Published:** 2014-04-17

**Authors:** Jakub Galgonek, Jiří Vondrášek

**Affiliations:** 1 Institute of Organic Chemistry and Biochemistry, Academy of Sciences of the Czech Republic, Flemingovo nam. 2, 166 10 Prague 6, Czech Republic

## Abstract

**Background:**

There are many databases of small molecules focused on different aspects of research and its applications. Some tasks may require integration of information from various databases. However, determining which entries from different databases represent the same compound is not straightforward. Integration can be based, for example, on automatically generated cross-reference links between entries. Another approach is to use the manually curated links stored directly in databases. This study employs well-established InChI identifiers to measure the consistency and completeness of the manually curated links by comparing them with the automatically generated ones.

**Results:**

We used two different tools to generate InChI identifiers and observed some ambiguities in their outputs. In part, these ambiguities were caused by indistinctness in interpretation of the structural data used. InChI identifiers were used successfully to find duplicate entries in databases. We found that the InChI inconsistencies in the manually curated links are very high (28.85% in the worst case). Even using a weaker definition of consistency, the measured values were very high in general. The completeness of the manually curated links was also very poor (only 93.8% in the best case) compared with that of the automatically generated links.

**Conclusions:**

We observed several problems with the InChI tools and the files used as their inputs. There are large gaps in the consistency and completeness of manually curated links if they are measured using InChI identifiers. However, inconsistency can be caused both by errors in manually curated links and the inherent limitations of the InChI method.

## Background

In the field of small molecule research, there are many specialized databases focused on different aspects of chemical research and its applications [1]. The fragmentation of databases is not a problem if users want to find certain information about a specific compound (or other molecular entity) of interest. However, performing some tasks may require integration of information from various databases. In general, such integration is not easy because different databases may not use the same compound names and may use different structures (e.g., different tautomeric forms) to represent the same compounds.

Users can select several approaches to handle this problem. Databases can be merged into one, or users can employ a service that performs cross-referencing between database entries [2-6]. Finally, users can take advantage of cross-reference links stored directly in databases. Indeed, database entries often contain links that refer to the same compounds in other databases. Such a link typically is stored as an identifier that is assigned to the entry that represents the same compound in other database. In some databases, these links can be manually curated.

Merged databases or cross-referencing services use an automatic procedure to identify the same compounds in different databases. In general, this procedure involves generating an unambiguous identifier of a compound independent of the tautomeric form that was used as the input. Typically, the input structure is normalized to obtain a so-called canonical tautomer. This tautomer is then serialized to an identifier, which can be based, for example, on SMILES codes [7,8]. The identifiers then can simply be compared to determine whether two database entries represent the same compound.

This kind of identifier has a small disadvantage. It contains data (e.g., multiplicity of bonds, hydrogen atom positions, etc.) that are specific for the canonical tautomer but not for the whole compound. This disadvantage can be overcome by using InChI identifiers. The algorithm that generates an InChI identifier internally employs various normalization rules to generate the identifier independent of the specific form of the compound used as the input. In addition, InChI identifiers express information in a way that does not prefer any tautomeric form.

It might seem that manual links must be of a better quality, because unlike automatically generated links, they are not affected by systematic errors. Nevertheless, manually curated databases can also contain errors [9,10]. In addition to the mentioned link consistency issue, another frequent type of error is duplicate entries.

The work described here employs InChI to identify entries. We examine how these identifiers can be used to find duplicities in databases and how to measure the consistency of manually curated cross-reference links and their InChI identifiers. And finally, we study the completeness of manually curated links by comparing them with the automatically generated ones.

Older work that employs own set of normalization rules has focused on identifying duplicities in databases [4]. Others have evaluated measurement of the link consistencies [11]. However, we examined other methods of consistency measurement and assessment of all possible sources of inconsistencies. Although InChI is considered to be very robust [12], we strove for a critical interpretation of the methods used and the results obtained. We utilized two different tools that can be used to assign identifiers, and we showed that there are ambiguities both in their outputs and in the interpretation of data inputs. We attempted to carefully assess which discrepancies can be caused by human error and which can be caused by the insufficiency of the methods used.

## Methods

### Database entries

In a database of small molecules, each entry represents one molecular entity, where a molecular entity can be defined as any constitutionally or isotopically distinct atom, molecule, ion, ion pair, radical, radical ion, complex, conformer, etc. identifiable as a separately distinguishable entity. The degree of precision necessary to describe a molecular entity depends on the context [13]. This means that when a database is constructed, it is important to define what is considered to be the same and what is considered to be different. In general, this is not an easy task. It can be useful to organize entities hierarchically, carefully defining the *subtype* and other relations. In the Chemical Entities of Biological Interest (ChEBI) database, for example, *D-serine zwitterion* [CHEBI:35247] is a tautomer of *D-serine* [CHEBI:16523], which is a subtype of *serine* [CHEBI:17822]. A hierarchically organized database also may contain the *common D-serine* entity which does not distinguish between the molecule’s tautomeric forms and for which D-serine [CHEBI:16523] and D-serine zwitterion [CHEBI:35247] are subtypes.

Ideally, a database entry should contain a description of all possible forms of the entity represented. Such a description would make it possible to distinguish entities from each other as well as to decide whether a given compound is an instance of an entity.

In practice, Structure-Data Files (SDF files) in V2000 format are widely used in most small molecule databases to describe the structure of an entity [14,15]. Unfortunately, SDF files cannot describe all entity types precisely. For example, an SDF file cannot describe all tautomeric forms of an entity in a single record; therefore, only one tautomeric form must be used to represent the whole compound. For example, the common form of D-serine and D-serine [CHEBI:16523] cannot be easily distinguished by their SDF files. Another disadvantage is that an SDF file expresses values for all SDF data fields, even those that are irrelevant. For example, the isotopic form of an element must always be specified, and it is impossible to indicate that isotopic forms of some elements can be disregarded. The only structural information that can be specified in an SDF file as (partially) undefined is stereochemistry information.

The limitations of SDF files become an issue mainly when a database is organized hierarchically. Many databases of small molecules collect and store experimental data from a specific area of interest. In this case, the correct interpretation of an SDF file is implicit. When an SDF file describes a compound used in a chemical experiment, we may assume that the entity being described includes the tautomeric form that is directly stored in the SDF file, as well as all other tautomeric forms of the compound. However, this interpretation is not suitable for all databases based on experimental data. For example, the PDBeChem database focuses on three-dimensional structures of compounds and thus must distinguish between tautomeric forms.

To use InChI identifiers to identify entities, we have to suppose that an entity does not describe only the specific tautomeric form represented by its SDF file but all possible tautomeric forms. We also must assume that no value from the mandatory SDF data fields is ignored. All default values have to be respected. We also use InChI identifiers as representations of entities stored in databases for which this interpretation is not natural. In any case, the measured results have to be interpreted with caution.

### A description of InChI identifiers

The following paragraphs briefly describe the structure and generation of InChI identifiers. Generating an InChI identifier involves three steps [16]:

1. *Normalization* removes all information that is not necessary for InChI construction. During this step, several types of normalization rules are applied. For example, salts and metals are disconnected, variable protonation is processed, tautomers are detected, etc.

2. *Canonicalization* generates the numbering of the atoms that do not depend on how the structure was initially drawn.

3. *Serialization* converts the obtained information into a string of characters that constitutes the InChI identifier.

The resulting InChI identifier expresses information hierarchically in so-called *layers* and *sub-layers*. Each layer describes a specific type of chemical properties of the entity described:

1. The *main* layer describes basic structural properties. The *formula* sub-layer describes the numbers and types of atoms. The connections between heavy atoms are described by the *connections* sub-layer (prefixed/c). Because positions of hydrogen atoms can differ in different tautomeric forms, hydrogen atoms are described by a special sub-layer, called *H-atoms* (prefixed/h).

2. The *charge* layer consists of the *charge* sub-layer (prefixed/q), which describes the charge of the entity after normalization, and the *protons* sub-layer (prefixed/p), which expresses the number of protons added (or removed) during normalization.

3. The *stereo* layer describes the stereochemistry of the entity. Double-bond stereochemistry is described by the *double bond* sub-layer (prefixed/b). Tetrahedral stereochemistry is described by the *tetrahedral* sub-layer (prefixed/t). A question mark is used to denote a case in which the conformation of a double bond or chiral center is not defined. The tetrahedral sub-layers of enantiomers have the same value. To distinguish between enantiomeric structures, the *inverted tetrahedral* sub-layer (prefixed/m) is used. The last stereochemistry sub-layer is called the *stereo type* (prefixed/s). It describes the requested type of stereochemistry. The value 1, which denotes absolute stereochemistry, is the only value allowed in standard InChI identifiers.

4. The *isotopic* layer is used when non-standard atomic isotopes are present. Isotopes are described in the *isotopic atoms* sub-layer (prefixed/i) and the *isotopic exchangeable H* sub-layer (prefixed/h). If taking isotopes into account causes changes in stereochemistry, the new stereochemistry is described here by sub-layers that have the same meaning as ones in the stereo layer.

**Figure 1:**
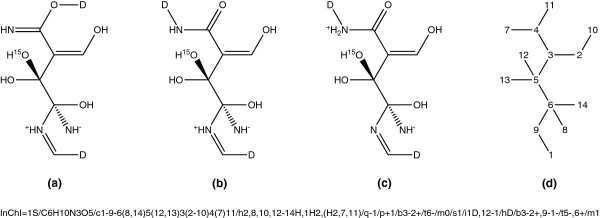
An example of an InChI identifier.

An example of an InChI identifier is provided in Figure 1. This example shows a hypothetical entity with a structure that employs all standard InChI sub-layers. In panels (a), (b), and (c), various tautomeric forms of the entity are shown. The canonical numbering of the heavy atoms is shown in panel (d).

**Figure 2:**
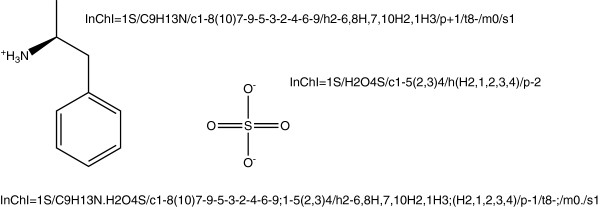
An example of a multicomponent InChI identifier.

Even if an entity contains multiple components, only one InChI identifier is generated. The parts that relate to the individual components are separated by dots in the formula sub-layer and by semicolons in the other sub-layers. The protons sub-layer is shared by all components and always contains only one number. An example of an InChI identifier describing multiple components is shown in Figure 2. The left side of the figure shows protonated (*S*)-amphetamine and its InChI identifier. The right side depicts sulfate and its InChI identifier. The InChI identifier of a mixture of these two compounds is shown at the bottom of the figure.

In a non-standard InChI identifier, additional layers may be included. The *fixed-hydrogen* layer describes fixed positions of mobile hydrogen atoms; therefore, it can be used to distinguish between some tautomers. Another non-standard layer can describe connections between disconnected components (metal atoms or salts are disconnected from the structure during normalization). Hereafter, we refer to an InChI identifier containing these additional layers as an *extended InChI identifier*.

SDF files of database entries must be converted to obtain InChI identifiers. We used two well-established tools – the inchi tool (version 1.04) and the molconvert tool (version 5.12.0). The inchi tool was developed by the International Union of Pure and Applied Chemistry (IUPAC) and the InChI Trust [17]. The molconvert tool is part of the JChem suite developed by ChemAxon [18]. The molconvert tool uses the inchi library 1.03 developed by IUPAC but its own SDF file parser. Both tools allow direct conversion of a given SDF file into the corresponding InChI identifier.

### Selection of chemical databases

For our experiments, we selected five specific databases – ChEBI [19], DrugBank [20], PDBeChem [21], Human Metabolome Database (HMDB) [22] and the NCGC Pharmaceutical Collection (NPC) [23]. ChEBI is a database of molecular entities focused on “small” chemical compounds. It incorporates an ontological classification whereby the relations between molecular entities are specified. DrugBank is a database that combines detailed drug (i.e., chemical, pharmacological and pharmaceutical) data with drug target (i.e., sequence, structure and pathway) information. PDBeChem is a database of ligands, small molecules and monomers referred to in Protein Data Bank (PDB) entries. HMDB contains detailed information about small molecule metabolites found in the human body. Finally, NPC is a database of approved and investigational drugs for high-throughput screening.

All of these databases support data download. For our purposes, the most important data are InChI identifiers and cross-reference links to other databases. ChEBI makes it possible to download data in various formats. We selected data stored in a relational database as Oracle binary table dumps. DrugBank allows the XML format and the DrugCard format. Although the DrugCard format has been deprecated, we noticed that the data stored in this format is more recent than the data stored in the XML format. Thus, we decided to use data stored in the DrugCard format. PDBeChem stores data in macromolecular Crystallographic Information Files (mmCIF), which is a subset of the Self-defining Text Archive and Retrieval (STAR) format [24]. HMDB uses the XML format. We ignored the files with names beginning with HMDB, considering them obsolete. Lastly, the NPC database allows users to download information about the entries in the Structure-Data File (SDF) format.

All of these data were downloaded on April 14, 2013. All of the selected databases were converted into Resource Description Framework (RDF) format [25-27] and have been merged into one database, which is based on the Oracle Database supporting the RDF Semantic Graph [28].

All of the selected databases also support the download of entity structures in the SDF file format. However, special treatment is needed in the case of the PDBeChem database. It describes the structures of entities using two different formats – SDF files and mmCIF files. Only SDF files can be used directly for the generation of an InChI identifier, but we observed some discrepancies between the information stored in these two file formats. An SDF file makes it possible to express only charges ranging from −3 to 3 in its atom block. If a charge falls outside of this range, an extension (in the form of the M CHG line included in the SDF properties block) should be used. However, the SDF files provided by the PDBeChem database do not include such extensions. For this reason, we developed our own conversion tools to convert mmCIF files into SDF files that support this extension. Moreover, since an mmCIF file contains hydrogen atoms explicitly, we set the valence field of each atom in the generated SDF file. This disables the possibility to add implicit hydrogen atoms using conversion tools. An mmCIF file contains two sets of atom coordinates – a representative set and an ideal set [21]. Each of these can be used to generate an SDF file. We selected the representative coordinates for our experiments (see Appendix A for details).

### Selection of entries

Not all database entries for which SDF files exist are suitable for assignment of InChI identifiers. An SDF file can describe a structure that contains undefined or general parts, e.g., an amino acid with a side chain denoted as an R group. These facts are usually described in special parts of an SDF file. Unfortunately, some of these parts are ignored by InChI conversion tools, resulting in generation of an incorrect InChI identifier. For this reason, we excluded entries with SDF files containing descriptions for which InChI identifiers should not be generated.

An entry can be assigned an InChI identifier if it has an SDF file describing the structure and all of the following conditions hold:

1. *It does not contain an element that is not an atom.* This is not common, but SDF files can contain non-atom elements (e.g., an electron or positron) or exotic atoms (e.g., muonium). These elements are not supported by InChI.

2. *It does not contain an unknown or general atom.* In addition to a symbol from the periodic table, an SDF file can also contain general elements, often denoted as A, Q, * or R.

3. *It does not contain an R-group.* Some atom of the structure may be aliased as an R-group, which indicates an undefined chemical group.

4. *It does not contain an undefined charge.* A valid SDF file cannot contain an undefined charge. However, if we convert an mmCIF file containing an undefined charge into an SDF file, the resulting SDF file is excluded.

5. *It does not contain an unknown type of bond.* This is not common, but an SDF file can contain an “any” bond, which does not specify the chemical type of the bond.

6. *It is not a polymer with an unknown number of repeating monomers.* Some part of the structure can be denoted as a repetitive part, but an InChI identifier cannot be generated if the number of repeats is not known.

### Searching for duplicates

Duplicate entries represent the same entity. Therefore, their SDF files contain the same structural information. However, the files might not be binary identical, and as a result, the SDF files cannot be directly compared to find duplicates. Nevertheless, the duplicate entries have the same InChI identifier assigned. In our search for duplicate entries, we therefore look for entries with the same InChI identifier. If two entries from one database have the same InChI identifier, we call it an *InChI collision*. However, not all InChI collisions are caused by duplicate entries, and we therefore have to define sources of InChI collisions.

There are three sources of InChI collisions:

1. The entries are actual duplicates.

2. The entities represented by the entries are different, but the SDF files cannot distinguish between them.

3. The entries can be distinguished by their SDF files, but the InChI identifier cannot distinguish between them.

The latter two possibilities represent false-positive cases. For this reason, we have defined an *extended InChI collision* as a pair of entries with the same extended InChI identifier, and an *SDF file collision* as a pair of entries with SDF files containing the same structural information. As stated, extended InChI identifiers can distinguish between entities that standard InChI identifiers cannot. An SDF file collision implies an extended InChI collision, and an extended InChI collision implies an InChI collision. Sets of colliding pairs can thus be organized as subsets, i.e., SDF file collisions are a subset of extended InChI collisions, which are a subset of InChI collisions.

As an SDF file collision does not imply that the SDF files of the colliding pair are binary identical, SDF file collisions have to be found manually. For that, however, it is enough to manually process only extended InChI collisions.

It is important to note that an SDF file collision does not imply duplicity in all cases. In a database, there can be a pair of entries that are different but that have the same SDF files.

### Comparison of InChI identifiers

If InChI identifiers are used to represent database entries, it is possible to simply compare the InChI identifiers to check whether the entries describe the same entity. However, different chemical databases may describe entities with different levels of generality. For this reason, the decision about whether two database entries represent the same entity is not flexible enough to determine whether a cross-reference link between two database entries is consistent. A better approach is to examine whether one of the linked entities is a subtype of the other entity.

In some cases, researchers have converted InChI identifiers back into structures [11]. We decided to compare InChI identifiers directly, because the back conversion is not fully reliable (see Appendix B for details).

Suppose that we want examine whether the entity described by InChI identifier X is a subtype of another entity described by InChI identifier Y. If so, identifier X must contain all the information that is contained in identifier Y. In this case, the identifiers are *compatible*, and identifier X is equally or *more specific* than identifier Y.

**Figure 3:**
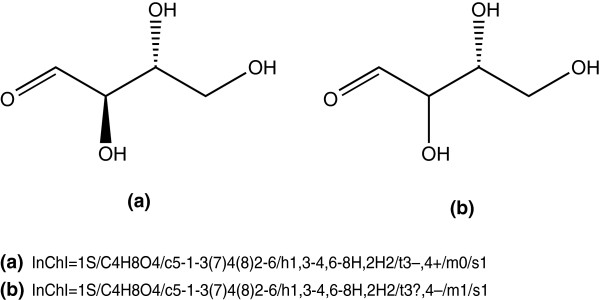
Influence of an undefined chiral center conformation on the inverted tetrahedral sub-layer.

First, we focus on InChI identifiers containing only one component. None of the InChI sub-layers, except for the stereochemistry sub-layers, can express that its value is (partially) unspecified. This means that these sub-layers have to be used in the same form in both compared identifiers. On the contrary, the stereochemistry sub-layers have to be processed by single parts. In the case of the double bond sub-layer if a conformation is defined in identifier Y, the same conformation must also be defined in identifier X. The same approach has to be applied to the tetrahedral sub-layers if the inverted tetrahedral sub-layers of both identifiers have the same value. If they do not, one of the tetrahedral sub-layers must be inverted before comparing. This special treatment is necessary because of the way enantiomers are described. As we have noted, if two entities are enantiomers, their tetrahedral stereochemistry sub-layers have the same values, and the entities are distinguished by inverted tetrahedral sub-layers. From two possible values of the tetrahedral sub-layer, the value that is smaller is selected. However, the selection can be altered when the conformation of a chiral center becomes undefined. Figure 3 illustrates an example of this situation. The structure in panel (a) has the conformations of both of its chiral centers defined. Panel (b) shows the same structure, but the conformation of the first chiral center is undefined. This change has caused an inversion of the tetrahedral stereochemistry sub-layers (prefixed/t).

To check the *compatibility* of multicomponent InChI identifiers, the sets of the components included in the InChI identifiers must be the same. This means that the entity described by InChI identifier X is a subtype of another entity described by InChI identifier Y if and only if both identifiers have the same number of components and the entity described by the n^th^ component of X is a subtype of the entity described by the n^th^ component of Y.

It is also possible to use a less strict approach for comparison of multicomponent InChI identifiers. This approach makes it possible to strip some components because it admits that an entity that is considered to be more specific can contain some additives. Only the first components of the InChI identifiers are considered to be the most significant. This approach thus considers the entity described by InChI identifier X as a subtype of the entity described by InChI identifier Y if and only if the entity described by the first component of X is a subtype of the entity described by the first component of Y and for each component L from Y there exists a component K from X such that the entity described by component K is a subtype of the entity described by component L. This approach is still sensitive to fragments. It allows linking of the neutral form of a compound with its salt, but it does not allow linking different salts together.

Both of the approaches described for comparison of multicomponent InChI identifiers require that a multicomponent InChI identifier be divided into multiple InChI identifiers describing individual components. From the definition of the InChI identifier, this is a simple task. The only sub-layer that cannot be unambiguously divided into components is the protons sub-layer because it contains one value summed over all components. For this reason, when a multicomponent InChI identifier is divided, only the first component obtains the value of the protons sub-layer from the original identifier. This is a formal decision to ensure that both of the original InChI identifiers have the same value of protons sub-layers independent of the way in which the identifiers are compared.

To make the link consistency check less strict, it is also possible to strip some types of information from the InChI identifiers before they are compared. There are several well-defined possibilities: removing the isotopes sub-layers, removing the protons sub-layer or removing the stereo sub-layers. If we want to perform only the most basic comparison of the structures of entities, it is appropriate to employ all these possibilities as well as to remove the information about hydrogen atoms and charges. This leads to preservation of the formula without hydrogen atoms and the connections sub-layer only.

Based on previous descriptions, we have defined different but related methods to check link consistency. In all cases, we utilized both proposed approaches to examine multicomponent InChI identifiers, i.e., the method that requires the same lists of components in the InChI identifiers being compared (denoted X⇔Y) and the method permitting the omission of some components (denoted X↔Y). Different approaches are then used to compare the InChI component. For the sake of comparison with other works, we tested InChI components for identity; this method is denoted CHSI, in which the letters denote the types of information checked for identity, namely: C – heavy atoms and their connections; H – hydrogen atoms, protonation and charge; S – stereochemistry; I – isotopic forms and related information. As stated, we do not consider this approach to be sufficient for consistency checking. The second approach utilized the above-described method for the comparison of the InChI component. We have denoted it CHsI because it does not strictly require the identity of the stereochemistry sub-layers. The subsequent method (denoted ChsI) additionally ignores the protonation of the InChI identifiers. Other methods are based on this method but ignore some additional InChI information. The method denoted Chs- ignores the isotopic layer while the method denoted Ch-I ignores the stereochemistry layer. The method denoted C**---** ignores all the information except for heavy-atom types and their connections.

### Automatic generation of cross-reference links

To measure the completeness of the links, we automatically generated cross-reference links between database entries and compared these links with manually curated links. In the first approach, we linked entries that represent the same entity, i.e., those with the same InChI identifier. Completeness of the manually curated links is then defined as the percentage of manually curated links included in the generated ones. For the sake of comparison, only manual links between entries with the same InChI identifiers were taken into the account.

We also tested another link-generation approach. When links are generated, it is more suitable to link an entity along with its subtypes. This is especially useful in situations when, for a given entity from a database, the other database does not contain the same entity. In our second approach, the entities are therefore linked if they are identified by compatible InChI identifiers. This approach is fully suitable for link generation, but some of the generated links may be considered redundant. This is an issue when the numbers of generated links are compared with the numbers of manually curated links. We attempted to link an entry from a database only with the most similar entries from another database. Other links, although consistent, are considered redundant.

We defined two rules to determine redundant links. According to the first, a link between entry X in database A and entry Y in database B is considered redundant if the following conditions hold:

1. Database A contains another entry X′ (different from X) with a link to entry Y.

2. The entities represented by X and X′ are equally or less specific than the entity represented by Y.

3. The entity represented by X′ is a subtype of the entity represented by X.

For example, if database A contains serine (X) and L-serine (X′) and database B contains L-serine (Y), then the link between serine (X) in database A and L-serine (Y) in database B is considered redundant.

According to the second rule, the link between entry X in database A and entry Y in database B is considered redundant if the following conditions hold:

1. Database A contains another entry X′ (different from X) with a link to entry Y.

2. The entities represented by X and X′ are equally or more specific than the entity represented by Y.

3. The entity represented by X is a subtype of the entity represented by X′.

For example, if database A contains serine (X′) and L-serine (X) and database B contains serine (Y), then the link between L-serine (X) in database A and serine (Y) in database B is considered redundant.

Consequently, if the other database contains entities with the same InChI identifiers as the entity in question, then the entity is linked only with them. It is thus linked with its indirect subtypes only if the other database does not contain the same entity.

## Results and discussion

### Assignment of InChI identifiers

The numbers of all the entries included in the databases are shown in Table 1 (the *entries in the database* column). The table also shows the numbers of entries with SDF files describing their structures (the *entries with structures* column) and the numbers of entries selected to be assigned InChI identifiers (the *entries selected* column).

**Table 1:** Number of entries in various databases

	**Entries**			**Converted by**		**Containing an ambiguous**	
**Database**	**In the database**	**With structures**	**Selected**	**Inchi**	**Molconvert**	**Center**	**Bond**
ChEBI	50500	29852	26502	26487	26494	678	17
DrugBank	6714	6516	6509	6403	6441	327	2
PDBeChem	15446	15445	15439	15439	15439	120	2
HMDB	40278	40233	40220	40219	40220	177	14
NPC	14814	8027	8013	8013	8001	95	1

Although we selected entries with SDF files that should be convertible into InChI identifiers, there were still files that could not be converted by at least one of the tools. Neither of the tools can convert an SDF file that contains an element with the symbol Uub, but this chemical symbol is considered to be deprecated. Moreover, molconvert cannot convert SDF files that contain an atom with a proton number greater than 104 because these atoms are not supported by inchi library 1.03. Other differences in the capabilities of the tools result from the different SDF parsers that they utilize. The inchi tool cannot convert SDF files that contain coordinated bonds because these bonds are written using an extension that is not supported by the inchi tool. Both tools also have problems with SDF files containing aromatic bonds unless they are written as alternating single and double bonds. Sometimes, a special SDF bond type is used to represent aromatic bonds, although it should not be used in such cases [15]. Each tool had problems with a different set of files containing this bond type. Finally, the molconvert tool fails to convert files containing misformatted attachment point (APO) lines. The inchi tool does not have problems with these lines because the information described by the lines is irrelevant for the generation of InChI identifiers.

The numbers of successfully converted SDF files are shown in Table 1 (in the columns *converted by inchi* and *converted by molconvert*). During the conversion, various warnings were reported. The warnings that are most relevant to our experiments indicate ambiguous stereochemistry descriptions. These warnings may explain some discrepancies in outputs of the tools. The numbers of SDF files for which an ambiguous conformation of a stereo center was reported are also shown in Table 1 (the column *containing an ambiguous center*). Similarly, the numbers of SDF files for which an ambiguous double bond conformation was reported are shown (the column *containing an ambiguous bond*). The numbers are based on the warnings produced by the inchi tool because relevant warnings produced by the molconvert tool were also produced by the inchi tool.

### Consistency of InChI identifier sources

**Figure 4:**
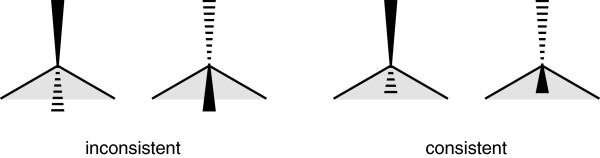
Inconsistencies in the interpretations of chiral center conformations.

Although both tools should generate the same outputs for a given input SDF file, there were some discrepancies. This is a serious problem because an InChI identifier is considered a unique identifier of an entity. We performed a careful analysis of all the discrepancies observed, and we have identified five classes (see Table 2):

1. The molconvert tool does not process a double-bond flag denoting that the Z/E stereoisomerism of the bond is undefined. In such cases, the molconvert tool determines the Z/E stereoisomerism based on the x, y coordinates of the substituents. The new information not included in the converted SDF files is thus added into the InChI identifier.

2. The molconvert tool does not fully support axial chirality. This kind of chirality is marked as undefined in the resulting InChI identifier.

3. There are many discrepancies in tetrahedral stereochemistry. In databases other than PDBeChem, which contains three-dimensional SDF files, an ambiguous stereo warning is reported in most cases when discrepancies arise. In two minor kinds of cases, no ambiguous stereo warning is reported. In the first case, if there is a chiral atom containing four explicit substituents – one connected by an up-bond and one connected by a down-bond on the opposite side of the drawing, then a discrepancy between the tools occurs if one of the substituents connected by the up- or down-bond is not located in the hypothetical triangle formed by the remaining atoms. An example is shown in Figure 4. In the second case, a discrepancy occurs when an up-bond or down-bond has a zero length in the drawing. In the PDBeChem database, discrepancies are caused by a non-realistic 3D position of the substituents. If we use the ideal coordinates of atoms to generate SDF files, the atoms have a chemically meaningful 3D position and only one discrepancy occurs, which is caused by an error in the data.

4. In some marginal cases, the tools use different rules to determine the default valences of the atoms. Note that the valence of an atom can be stored explicitly in an SDF file. However, this is not preferred by the authors of the databases.

5. Discrepancies may also arise from poor interpretation of aromatic bonds by the molconvert tool. However, this type of bond should not be included in a standard SDF data file [15]. Therefore, this class can be interpreted as a problem of input SDF files.

**Table 2:** Inconsistencies between the inchi tool and the molconvert tool

	**Inconsistency classes**						
**Database**	**Misused double-bond stereo**	**Unsupported stereo types**	**Ambiguous stereo (reported)**	**Ambiguous stereo (unreported)**	**Default valence interpretation**	**Aromatic bond interpretation**	**Total number of differences**
ChEBI	537	9	68	3	19	0	636
DrugBank	30	0	8	0	0	5	43
PDBeChem	0	0	27	68	0	0	95
HMDB	134	1	99	6	8	0	248
NPC	395	0	16	12	9	0	431

Discrepancies of the first two classes are caused by the insufficiencies of the molconvert tool. However, a bigger problem is that the remaining types of discrepancies can be interpreted as ambiguities in the interpretation of input SDF files.

For the sake of completeness, we also compared the InChI identifiers stored in the databases with the identifiers generated by the tools. Because the NPC database does not include InChI identifiers, we excluded it from these measurements. The numbers of differences are listed in Table 3 (the *different* columns). The table also includes the numbers of entries that have InChI identifiers stored in a database but for which the InChI identifiers cannot be generated from SDF files (the *extra in the database* columns). The main reasons for this are the exclusion of several SDF files (see Selection of entries) and the insufficiencies of the tools used (see Assignment of InChI identifiers).

**Table 3:** Inconsistencies between database InChI identifiers and identifiers generated by the tools

	**Inchi**			**Molconvert**		
**Database**	**Extra in the database**	**Missing in the database**	**Different**	**Extra in the database**	**Missing in the database**	**Different**
ChEBI	154	24	0	154	31	636
DrugBank	47	2	45	7	0	2
PDBeChem	4	539	54	4	539	146
HMDB	39	0	243	38	0	5

Conversely, the table also includes the numbers of entries that have no InChI identifiers stored in a database but for which InChI identifiers can be generated from SDF files (the *missing in the database* columns). Non-standard InChI identifiers (i.e., identifiers having a different version than 1S) stored in the PDBeChem database are not taken into account.

The table shows that the ChEBI database uses the inchi tool, as does the PDBeChem database. Nevertheless, there are several discrepancies in the case of the PDBeChem database. Careful analysis implied that these discrepancies may have been caused by the obsolete data used to generate InChI identifiers stored in the database. In many cases, for example, the InChI identifier stored in an entry indicates a different number of atoms or a different charge than a recent structure described by the entry.

The rest of the databases that contain InChI identifiers, i.e., the DrugBank and HMDB databases, use the molconvert tool. The number of discrepancies is very low. In the case of the DrugBank database, we found only two discrepancies. Their InChI identifiers stored in the database are equal to the output of the inchi tool. We found five discrepancies in the HMDB database, but the tools are consistent in their interpretations of these five entries.

For subsequent experiments (results below), we used InChI identifiers generated by the inchi tool. As shown above, the input SDF file can be ambiguous in some cases; therefore, the InChI identifiers generated may not be fully reliable. Nevertheless, if we exclude the SDF files for which a warning of ambiguity is reported or for which different InChI identifiers are generated by the tools, the effects on the results of the experiments performed will be marginal.

### Duplicate entries

Numerous kinds of collisions were found in each of the selected databases. The numbers are shown in Table 4. The ChEBI database describes most of the chemical elements twice – as descendants of the entry *atom* [CHEBI:33250] and as descendants of the entry *molecular entity* [CHEBI:23367]. Consequently, the descendants of the atom [CHEBI:33250] entry were excluded from this experiment.

**Table 4:** Numbers of collisions

	**Collisions**		
**Database**	**InChI**	**Extended InChI**	**SDF file**
ChEBI	459	62	32
DrugBank	145	143	142
PDBeChem	74	48	34
HMDB	39	39	39
NPC	42	42	42

Different databases require different approaches to find duplicities. For the DrugBank, HMDB and NPC databases, InChI collisions are a good way to identify duplicities because the use of InChI identifiers is natural for these databases. On the other hand, for the PDBeChem database, it is more convenient to use SDF file collisions because this database is focused on the structure, and thus it distinguishes between different tautomeric forms. To find duplicities in the ChEBI database, it is also convenient to use SDF file collisions. However, in this case, some entities cannot be distinguished by the SDF files. We performed a manual revision of all SDF file collisions in the ChEBI database and identified only 23 pairs as true duplicities.

In 2008, Sitzmann et al. showed that ChEBI contains 5.4% duplicates [4]. Their result was based on tautomer-insensitive comparison of structures. Although the authors used different tautomeric rules, their results can be roughly compared with results from searching InChI collisions. For the ChEBI database, we found 1.7% duplicates by searching InChI collisions. If additional tautomeric rules are used (see Appendix C for details), there are 3.1% duplicates observed by InChI collisions. Similarly, for the DrugBank database, Sitzmann et al. found 7.4% duplicates. We found 1.8% duplicates by InChI collisions; 2.1% if the additional tautomeric rules are used.

### Database link consistency

The numbers of cross-reference links obtained from the databases are shown in Table 5 (the *links* column). If database A contains links to database B, we refer to database A as a *source database* and to database B as a *target database*. This kind of cross-reference link is then denoted A → B. Not all the obtained links can be considered valid because some of them refer to entries that no longer exist in the target databases. The numbers of valid links can be seen in the same table. The large difference between the numbers of links and the numbers of valid links from the HMDB database to the ChEBI database is caused by the fact that the HMDB database labels ChEMBL identifiers as ChEBI identifiers. A link can be checked using our methods only if the link is valid and both linked entries have InChI identifiers assigned. The numbers of these links are shown in the last column (called *links with InChI*) of the table.

**Table 5:** Numbers of cross-reference links

**Type of links**	**Links**	**Valid links**	**Links with InChI**
HMDB → PDBeChem	1247	1192	1188
HMDB → DrugBank	1601	1589	1546
HMDB → ChEBI	4795	3848	3727
ChEBI → PDBeChem	2112	2020	1950
ChEBI → DrugBank	2462	2459	2336
ChEBI → HMDB	729	725	721
DrugBank → PDBeChem	5201	5153	5029
DrugBank → ChEBI	1826	1808	1724
NPC → DrugBank	1340	1340	1333

In our experiment, we used all the comparison methods described in the section Comparison of InChI identifiers. The results are shown in Table 6. The numbers indicate the fraction of the links that are considered to be inconsistent when the selected method is used. The numbers of the checked links (that form 100%) are in the last column of Table 5.

**Table 6:** Inconsistencies in cross-reference links

	**X⇔Y**						**X↔Y**					
**Type of links**	**CHSI**	**CHsI**	**ChsI**	**Chs-**	**Ch-I**	**C---**	**CHSI**	**CHsI**	**ChsI**	**Chs-**	**Ch-I**	**C---**
HMDB → PDBeChem	1.26%	0.93%	0.93%	0.93%	0.84%	0.59%	1.26%	0.93%	0.93%	0.93%	0.84%	0.59%
HMDB → DrugBank	7.12%	5.11%	4.46%	4.46%	1.88%	1.55%	7.05%	5.05%	4.40%	4.40%	1.81%	1.49%
HMDB → ChEBI	13.84%	7.06%	6.79%	6.79%	4.51%	3.33%	13.71%	6.92%	6.63%	6.63%	4.29%	3.09%
ChEBI → PDBeChem	9.64%	8.21%	6.51%	6.51%	4.62%	1.69%	9.64%	8.21%	6.46%	6.46%	4.56%	1.54%
ChEBI → DrugBank	33.18%	28.85%	27.95%	27.91%	18.96%	17.85%	22.99%	17.77%	13.74%	13.70%	4.54%	3.25%
ChEBI → HMDB	25.80%	10.40%	6.38%	6.38%	3.05%	2.91%	25.10%	9.71%	5.55%	5.55%	2.22%	2.08%
DrugBank → PDBeChem	27.04%	25.49%	25.17%	25.17%	4.04%	2.11%	27.02%	25.47%	25.13%	25.13%	4.00%	2.01%
DrugBank → ChEBI	18.33%	15.08%	13.98%	13.98%	3.13%	1.91%	18.10%	14.85%	13.69%	13.69%	2.84%	1.57%
NPC → DrugBank	23.26%	13.13%	12.90%	12.90%	6.90%	5.63%	22.73%	12.45%	11.25%	11.25%	4.65%	3.15%

First, we focused on the CHSI/X⇔Y method, in which a link is consistent if and only if the linked entries have the same InChI identifiers. When this method was used, the link inconsistency was high in most cases tested; the values varied from 1.26% to 33.18%. The most inconsistent links were those from ChEBI to DrugBank and HMDB, from DrugBank to PDBeChem and ChEBI and from NPC to DrugBank. In all of these cases, the inconsistency was greater than 18%.

If we allowed that one of the entities compared does not have to contain all the components of the other entity (i.e., the CHSI/X↔Y method was used), this had the greatest influence on the links from ChEBI to DrugBank – the value of inconsistency decreased from 33.18% to 22.99%. The influence on the other kinds of links was relatively marginal.

A better approach is to use methods that respect the fact that the linked entities do not have to be defined on the same level of generalization. If the only requirement was that the linked entities have compatible InChI identifiers (i.e., the CHsI/X⇔Y method was used), the values of inconsistency dropped significantly in many cases. Even better results were obtained if some components may be omitted (i.e., the CHsI/X↔Y method was used). Other acceptable methods are the ChsI/X⇔Y method and the ChsI/X↔Y method, which additionally ignore the protonation of the entities compared. When the ChsI/X↔Y method was used, the values of inconsistency are much better than in cases in which the identity of the InChI component is required. However, the inconsistencies in the links from ChEBI to DrugBank, from DrugBank to PDBeChem and ChEBI and from NPC to DrugBank remained high – above 10%.

As a basis for identification of the sources of these inconsistencies, the ChsI method was used, and some additional information was ignored. Methods that ignore the isotopic information are denoted as Chs-. As can be seen from Table 6, this had only a marginal influence.

The effect of ignoring the stereochemistry information (Ch-I methods) was much greater. A comparison with ChsI methods showed that many kinds of links do not use stereochemistry information. When the Ch-I/X↔Y method was used, all inconsistencies were below 5%.

Last methods, denoted C**---**, require only basic consistency between linked entries. When the C**---**/X↔Y method was used, the values of inconsistency varied between 0.59% and 3.25%. Although this last check is too lenient, it shows that most of the links have a proper basis.

**Figure 5:**
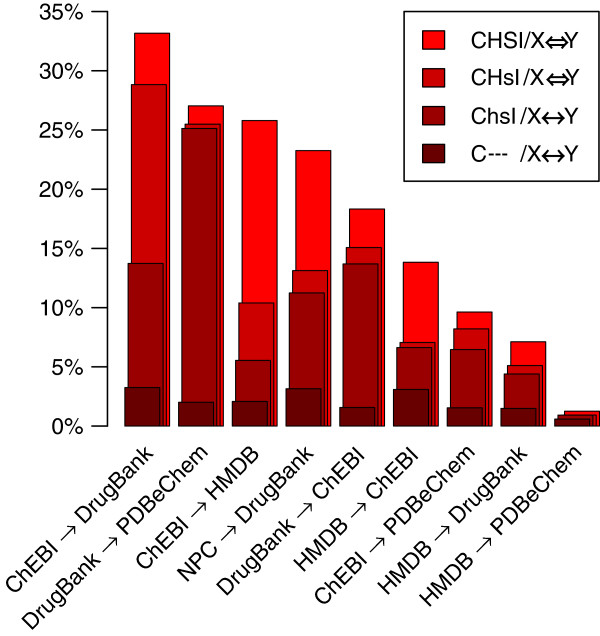
Inconsistencies in cross-reference links.

For better illustration, the results of the methods selected are also presented in a graphical format (Figure 5). The CHSI/X⇔Y method, in which the InChI identifiers compared have to be identical, is the most strict. On the other hand, the C**---**/X↔Y method is the least stringent of our methods. If all InChI components have to be taken into account, it is best to use the CHsI/X⇔Y method, which only requires that InChI identifiers be compatible and which does not ignore any information. When some of the InChI components can be omitted, it is appropriate to use the ChsI/X↔Y method, which – unlike the previous method – ignores the protonation of the entity, because the protonation cannot be unambiguously interpreted in a multicomponent InChI identifier.

Although the measured inconsistency is very high, an improvement can be seen if we compare our measurements with previously published ones [11]. When the InChI identity is measured (i.e., the CHSI/X⇔Y method is used), inconsistency decreases distinctly in many cases. For example, the inconsistency of links from HMDB to ChEBI decreases from 36.0% to 13.8%. Both measurements show that ignoring stereochemistry has a great positive influence on the measured inconsistencies in general. Nevertheless, the inconsistency of links from ChEBI to DrugBank remains high in both measurements.

Not all observed inconsistencies indicate that the appropriate links are incorrect. The InChI algorithm has its limitations; the different tautomeric forms of the same compound can be assigned different InChI identifiers in some cases [16,29]. Using additional tautomeric rules (see Appendix C for details) decreases measured inconsistencies. Nevertheless, we assume that links that are inconsistent by the C**---**/X↔Y method are actually incorrect.

### Completeness of manually curated links

First, we focused on links between entries with the same InChI identifiers. The numbers of generated links and manually curated links fulfilling this condition are shown in Table 7 in the section named *links between the same entities*. The completeness of the links is presented in the same section. Since the links between the ChEBI database and the DrugBank database are contained in both databases, we merged these two sets of links into one. We did the same for links between the ChEBI database and the HMDB database. The results for the merged links are shown in the last two rows of the table.

**Table 7:** Completeness of manually curated links

	**Links between the same entities**			**Links between compatible entities**		
**Type of links**	**Automatically generated**	**Manually curated**	**Completeness**	**Automatically generated**	**Manually curated**	**Completeness**
HMDB → PDBeChem	1251	1173	93.8%	1613	1177	73.0%
HMDB → DrugBank	1771	1436	81.1%	1900	1467	77.2%
HMDB → ChEBI	3634	3211	88.4%	4584	3464	75.6%
ChEBI → PDBeChem	2773	1737	62.6%	2961	1765	59.6%
ChEBI → DrugBank	1989	1561	78.5%	2078	1662	80.0%
ChEBI → HMDB	3634	535	14.7%	4584	646	14.1%
DrugBank → PDBeChem	3938	3669	93.2%	4073	3747	92.0%
DrugBank → ChEBI	1989	1408	70.8%	2078	1464	70.5%
NPC → DrugBank	1299	1021	78.6%	1480	1155	78.0%
ChEBI → DrugBank	1989	1563	78.6%	2078	1665	80.1%
ChEBI → HMDB	3634	3322	91.4%	4584	3653	79.7%

We found that the completeness of the manually curated links is not very high. The links from the ChEBI database to the HMDB database were the least complete (14.7%). We observed the best completeness for links from the HMDB database to the PDBeChem database (93.8%) and for links from the DrugBank database to the PDBeChem database (93.2%).

In the second approach, non-redundant links between entries with compatible InChI identifiers were generated. The results are shown in Table 7 in the section named *links between the compatible entities*. In this approach as well, the completeness of the manually curated links was poor. As we found using the first approach, links from the ChEBI database to the HMDB database were the least complete (14.1%). The completeness of links from the HMDB database to the PDBeChem database has decreased to only 73.0%. We observed the best completeness for links from the DrugBank database to the PDBeChem database (92.0%).

## Conclusions

We tested the ChEBI, DrugBank, PDBeChem, HMDB and NPC databases to measure the quality of cross-reference links. We observed many ambiguities both in the outputs of the tools generating InChI identifiers and in interpretation of the data. We showed that InChI identifiers can be used to find duplicities in chemical databases, but manual checking is still necessary because InChI identifiers cannot distinguish between all kinds of molecular entities. Like other investigators, we found that a large part of linked entries have different InChI identifiers and that many of the differences are located in the stereochemistry layers. We introduced a new method for testing link consistency based on the idea that the linked entities do not have to be identical – it is sufficient if one of the linked entities is a subtype of the other. Our method revealed that the link consistency is better than it seemed initially, but it is still relatively low. Finally, we measured the completeness of manually curated links by comparing them with automatically generated links. We observed gaps in the completeness of the links as well.

## Appendixes

### Appendix A: Consistency of PDBeChem InChI identifiers

The PDBeChem database contains two sets of coordinates – representative coordinates and ideal coordinates. Representative coordinates are manually chosen from all the occurrences of the ligand in PDB files. Ideal coordinates are automatically generated by the CORINA software package. They are not based on experimental data but on the molecule connectivity, bond orders and chirality to produce a molecular conformation that is energetically favorable in isolation and visually elegant in 3-D space [21].

InChI identifiers generated from these sets in theory should be the same. However, we noticed many differences. When the inchi tool was used, there were 441 entries with different InChI identifiers. There were 402 entries with different InChI identifiers when the molconvert tool was used. All these differences were located in the stereochemistry layers.

We decided to use the representative coordinates because the InChI identifiers generated from these coordinates are in better agreement with the InChI identifiers stored in the database. There were only 54 differences when the inchi tool was used with representative coordinates. On the other hand, there were 443 differences when the inchi tool was used with ideal coordinates.

### Appendix B: Back conversion of InChI to structure

An InChI identifier can be converted back into an SDF file. In some experiments, the possibility of converting an InChI identifier back into a structure has been used to perform additional normalization steps executable by other tools working with SDF files [11]. An example of an additional step is the removal of small fragments. After the normalization steps, the new SDF file is converted into an InChI identifier.

As each InChI identifier represents a set of structures, the result of the back conversion may not be the same as the SDF file that was used to generate the InChI identifier. Nevertheless, when the resulting SDF file is again converted into the InChI identifier, this identifier should be the same as the identifier used at the beginning.

To measure the quality of back conversion, all standard InChI identifiers stored in the selected databases were selected. These 76,300 identifiers were converted into SDF files, and the files were then converted back into InChI identifiers.

Since the inchi tool cannot restore stereochemistry information during back conversion, we compared the original and new InChI identifiers in two modes. In the first mode, the whole InChI identifiers were compared. In the second mode, the InChI identifiers were compared after the stereochemistry sub-layers were stripped.

The numbers of discrepancies are shown in Table 8. When using the molconvert tool, we also took the possibility to perform direct conversion. In this approach, an InChI identifier is directly converted into a new InChI identifier, so no temporary SDF file is used.

**Table 8:** Number of errors in the back conversions of InChI identifiers

**Method**	**Non-identity**	**Non-identity after stripped stereochemistry**
Inchi	N/A	97
Molconvert	1559	601
Molconvert-direct	773	595

The results show that the back conversion is not fully reliable. The number of errors produced by the inchi tool was relatively small; unfortunately, however, this tool does not restore stereochemistry information. For this reason, we avoided using back conversion in our experiments.

### Appendix C: Additional tautomeric rules

The inchi tools offer the possibility of employing additional tautomeric rules to handle keto-enol tautomerism and 1,5-tautomerism. These rules were introduced in a later version of InChI, and they are disabled by default [29]. Because these additional rules can have significant impact on the results, we repeated our main experiments with these rules enabled.

The revised number of collisions is shown in Table 9. The rules only influence the InChI collisions because extended InChI identifiers (and SDF files) distinguish between tautomeric forms. The significant increase in InChI collisions in the ChEBI database was expected because the database stores tautomeric forms as different entities.

**Table 9:** Additional rules: the number of collisions

	**Collisions**		
**Database**	**InChI**	**Extended InChI**	**SDF file**
ChEBI	1440	62	32
DrugBank	162	143	142
PDBeChem	175	48	34
HMDB	98	39	39
NPC	50	42	42

In general, the additional rules had a significant positive effect on the consistencies of the links, as shown in Table 10. This indicates that the selection of the normalization rules has a significant impact on the measurements, as has been previously suggested [4]. Nevertheless, the overall pattern of results remains the same.

**Table 10:** Additional rules: the inconsistencies of cross-reference links

	**X⇔Y**						**X↔Y**					
**Type of links**	**CHSI**	**CHsI**	**ChsI**	**Chs-**	**Ch-I**	**C---**	**CHSI**	**CHsI**	**ChsI**	**Chs-**	**Ch-I**	**C---**
HMDB → PDBeChem	1.26%	0.93%	0.93%	0.93%	0.84%	0.59%	1.26%	0.93%	0.93%	0.93%	0.84%	0.59%
HMDB → DrugBank	6.86%	4.92%	4.27%	4.27%	1.81%	1.55%	6.79%	4.85%	4.20%	4.20%	1.75%	1.49%
HMDB → ChEBI	12.07%	6.82%	6.55%	6.55%	4.40%	3.33%	11.94%	6.68%	6.39%	6.39%	4.19%	3.09%
ChEBI → PDBeChem	9.28%	8.05%	6.26%	6.26%	4.46%	1.69%	9.28%	8.05%	6.21%	6.21%	4.41%	1.54%
ChEBI → DrugBank	32.02%	28.21%	27.23%	27.18%	18.84%	17.85%	21.66%	17.08%	12.97%	12.93%	4.41%	3.25%
ChEBI → HMDB	22.19%	10.26%	6.24%	6.24%	3.05%	2.91%	21.50%	9.57%	5.41%	5.41%	2.22%	2.08%
DrugBank → PDBeChem	24.78%	23.34%	23.03%	23.03%	3.92%	2.11%	24.76%	23.32%	22.97%	22.97%	3.86%	2.01%
DrugBank → ChEBI	17.29%	14.50%	13.34%	13.34%	3.13%	1.91%	17.05%	14.27%	13.05%	13.05%	2.84%	1.57%
NPC → DrugBank	20.63%	11.48%	11.25%	11.25%	5.93%	5.63%	20.11%	10.80%	9.60%	9.60%	3.68%	3.15%

As shown in Table 11, the additional rules also had a significant positive impact on the numbers of links generated. The influence on the numbers of manually curated links used for the comparisons was less pronounced. Overall, the completeness was lower. For example, in the case of linking the same entities, the completeness of the links from HMDB to PDBeChem decreased from 93.8% to 85.2% when the additional rules were implemented.

**Table 11:** Additional rules: the completeness of manually curated links

	**Links between the same entities**			**Links between compatible entities**		
**Type of links**	**Automatically generated**	**Manually curated**	**Completeness**	**Automatically generated**	**Manually curated**	**Completeness**
HMDB → PDBeChem	1376	1173	85.2%	1700	1177	69.2%
HMDB → DrugBank	1825	1440	78.9%	1944	1470	756%
HMDB → ChEBI	4076	3277	80.4%	4935	3473	70.4%
ChEBI → PDBeChem	3217	1744	54.2%	3389	1768	52.2%
ChEBI → DrugBank	2173	1588	73.1%	2254	1677	74.4%
ChEBI → HMDB	4076	561	13.8%	4935	647	13.1%
DrugBank → PDBeChem	4102	3783	92.2%	4229	3855	91.2%
DrugBank → ChEBI	2173	1426	65.6%	2254	1474	65.4%
NPC → DrugBank	1359	1056	77.7%	1524	1177	77.2%
ChEBI → DrugBank	2173	1590	73.2%	2254	1680	74.5%
ChEBI → HMDB	4076	3391	83.2%	4935	3662	74.2%

## Abbreviations

ChEBI: Chemical entities of biological interest; HMDB: Human metabolome database; IUPAC: International union of pure and applied chemistry; mmCIF: Macromolecular crystallographic information file; NPC: NCGC pharmaceutical collection; PDB: Protein data bank; RDF: Resource description framework; SDF: Structure-data file; STAR: Self-defining text archive and retrieval.

## Competing interests

The authors declare no competing interests.

## Authors’ contributions

JG conceived the methods, processed and analyzed the data, and drafted the manuscript. JV supervised the project and revised the manuscript. Both authors read and approved the final manuscript.
